# Hydrotherapy in Pain Management in Pregnant Women: A Meta-Analysis of Randomized Clinical Trials

**DOI:** 10.3390/jcm13113260

**Published:** 2024-05-31

**Authors:** Elena Mellado-García, Lourdes Díaz-Rodríguez, Jonathan Cortés-Martín, Juan Carlos Sánchez-García, Beatriz Piqueras-Sola, María Montserrat Prieto Franganillo, Raquel Rodríguez-Blanque

**Affiliations:** 1 Department of Nursing, Faculty of Health Sciences, University of Granada, 18016 Granada, Spain; e.elenamellado@go.ugr.es (E.M.-G.); cldiaz@ugr.es (L.D.-R.); jcortesmartin@ugr.es (J.C.-M.); rarobladoc@ugr.es (R.R.-B.); 2 Virgen de las Nieves University Hospital, 18014 Granada, Spain; beatriz.piqueras.sspa@juntadeandalucia.es; 3 San Cecilio University Clinical Hospital, 18016 Granada, Spain; montseprietof@gmail.com

**Keywords:** hydrotherapy, waterbirth, immersion, first labor stage, neonatal health, maternal health

## Abstract

**Background**: the benefits of water are significant during the birth process. Improved maternal experience of labor, less use of epidurals, better pain management, shorter labor, and a greater sense of control are observed during the birth process. **Objective**: This report aims to determine the benefits of hydrotherapy in clinical childbirth approaches and its applicability in pain control. **Methods:** A meta-analysis of randomized clinical trials selected from various databases with no publication date limits was conducted, comparing groups that did not use hydrotherapy with groups that did during labor. **Results:** Seven articles met the inclusion criteria, with five articles using hot water immersion and two using hot water shower as hydrotherapy treatments. This study identified 840 participants, with the intervention groups including 417 term pregnant women and the control groups including 423 pregnant women. The effect size of hydrotherapy on pain was calculated using the visual analog scale in five articles and analgesic use in the other two articles. Hydrotherapy significantly reduced pain during labor with a mean difference of −0.97 (95% CI: −1.91 to −0.03; I^2^ = 97.32%, *p* < 0.001). The duration of the first stage of labor was not significantly affected, with a mean difference of −0.17 h (95% CI: −0.55 to 0.21; I^2^ = 56.75%, *p* = 0.059). Additionally, hydrotherapy did not significantly impact the newborns’ Apgar scores at 5 min, with a mean difference of 0.18 (95% CI: −0.48 to 0.85; I^2^ = 2.15%, *p* = 0.939). **Conclusions:** Hydrotherapy is beneficial for pain control in the first stage of labor and does not increase its duration or negatively affect the Apgar score of newborns.

## 1. Introduction

The use of water as a therapeutic medium has ancient origins, with evidence showing its use in China, Egypt, Japan, Greece, and Rome for treating physical and psychological ailments. In their literature review, Cluett, Burns, and Cuthbert report on the existence of historical references documenting the use of water immersion during childbirth for the purpose of achieving relaxation and pain relief [1]. Hydrotherapy during childbirth focuses on the comfort and support of pregnant women, and many find this method beneficial [2]. Water can be used during the first stage of labor (dilation), the second stage (expulsion), or both. In Spain, the Clinical Practice Guideline on Normal Childbirth recommends warm water immersion as an effective pain relief method during the active first stage of labor [3].

This method has several key features that make it an attractive option. Hydrotherapy is primarily used during the first stage of labor, when contractions are most intense and cervical dilation is in progress. The water temperature is typically maintained around 37.4 °C, which is comfortable for the mother and safe for the newborn. The water helps reduce pain and stress and can accelerate the dilation process. Warm water relaxes the muscles, reduces the perception of pain, and decreases the need for epidural analgesia. Additionally, the buoyancy of the water allows women to move more freely and adopt more comfortable positions during labor, which can relieve pressure in certain areas of the body. Moreover, the reduced gravity and abdominal pressure facilitate fetal rotation and descent, providing further advantages of hydrotherapy [4,5].

Defining the stages of labor is essential: the first stage (the latent phase from the beginning to 4 cm of cervical dilation and the active phase from 4 cm to 10 cm of cervical dilation), the second stage (expulsion), and the third stage (delivery of the placenta). Proper definition helps differentiate the maternal and neonatal risks and benefits of hydrotherapy [3].

A woman should give birth in a place where she feels secure and receives appropriate care (International Federation of Gynecology and Obstetrics, 1982). Hydrotherapy can enhance the childbirth experience and maternal health, as indicated by a qualitative study involving 23 women [6]. The benefits of water, such as buoyancy, hydrostatic pressure, and temperature, positively affect the dilation process. Studies report reduced epidurals use, better pain management, and shorter labor durations [7,8,9]. A systematic review and meta-analysis by Burns et al. (2022) [10] indicated a trend favoring water immersion for pain relief since 2009.

A Cochrane review of 15 trials involving 3663 women [1] compared water immersion with non-immersion. Eight studies involved water immersion during the first stage, and four involved it during both stages. The review found physical and emotional benefits [1], including higher pain thresholds, shorter dilation stages, reduced medical intervention, improved relaxation, and greater overall satisfaction with childbirth [11].

A cross-sectional study at São Bernardo Hospital in Portugal evaluated maternal and neonatal outcomes during labor stages. Excellent Apgar scores and pain relief were reported by 98.9% of the 90 women, with immersion time influencing labor duration significantly [12].

Despite these findings, some associations, such as the American College of Obstetricians and Gynecologists (ACOG) and the American Academy of Pediatrics (AAP) [13], discuss neonatal outcomes and safety, emphasizing the need for more high-quality studies.

Contrary to these concerns, Burns et al.’s meta-analysis [10] reported clear benefits for women and newborns from hydrotherapy, with no worse outcomes for water births. Other studies compare births with and without hydrotherapy and do not suggest worse outcomes for babies born through water birth [7,14]. The American College of Nurse-Midwives (ACNM) recommends providing evidence-based information on water birth for uncomplicated pregnancies [7].

A systematic review by Jacoby et al. [15] found varying perinatal outcomes for hydrotherapy, highlighting the need for further research. Meta-analyses and reviews of observational studies, including over 30,000 births, do not demonstrate increased risks for mothers or babies.

This meta-analysis aims to address pain management during the first stage of labor using minimally invasive techniques, enhancing healthcare quality and supporting the use of hydrotherapy for its beneficial impact on labor times and safety.

### Objectives

The primary objective is to determine the benefits of hydrotherapy in clinical childbirth approaches and its applicability in pain control. The secondary objectives include assessing its impact on the duration of the first stage of labor and the newborns’ physical condition.

## 2. Materials and Methods

### 2.1. Review Protocol

This systematic review and meta-analysis followed the PRISMA protocol and was registered with PROSPERO (CRD42023399625).

### 2.2. Search Strategy and Inclusion Criteria

Studies were selected based on the PICOS criteria (participants, interventions, comparisons, outcomes, and study design). Articles using the RCT methodology and involving pregnant women in the first stage of labor receiving hydrotherapy treatment were included. Two of the investigators (J.C.S.-G. and E.M.-G.) searched the Scopus, PubMed, Cinahl, and WOS databases. A manual search was also performed using the reference lists of studies to find other relevant research.

The structured language used was obtained using MeSH terms and Health Sciences (DeCS) descriptors. The descriptors used were “labor stage, first” and “immersion” along with the corresponding natural language descriptors, using the Boolean operator AND. Supplementary Table S1 shows the search strategy employed for each of the databases consulted, along with the dates on which the searches were conducted. The searches were performed without a year filter to obtain all relevant information related to the objective of the search. The articles were collected between December 2022 and January 2023.

### 2.3. Data Extraction and Quality Assessment

After carrying out the search strategy, the articles found were transferred to the Mendeley web application using the Mendeley web importer tool. They were then organized into folders according to the database from which they were obtained, and all duplicates were removed. The included studies were RCTs that met the objective of the search. Two reviewers (J.C.S.-G. and E.M.-G.) independently examined the title, abstract, and keywords of each study identified in the search and applied the inclusion and exclusion criteria. The same procedure was applied to potentially eligible full-text articles. Differences between reviewers were resolved by discussion or by a third reviewer (R.R.-B.).

Data on the quality, patient characteristics, interventions, and relevant outcomes were extracted independently by two reviewers (E.M.-G. and J.C.-M.).

Two reviewers (J.C.S.-G. and E.M.-G.) independently extracted the following data from each article: author, country and methodology of the study; intervention characteristics; sample size and sample distribution; weeks of gestation; sample selection criteria; and mean age. Regarding the results of the RCTs, we extracted the type of intervention, start of intervention, and duration of intervention, pain scale; furthermore, relative to the newborn, we assessed their physical condition at 5 min after birth. These data are reported in Table 1. The reviewers also assessed the strengths and weaknesses of each RCT.

A methodological quality assessment was performed using the PEDro (Physiotherapy Evidence Database) scale, as the methodology corresponded to RCTs. Publication bias was determined by visual inspection of the funnel plots.

### 2.4. Statistical Analysis

Statistical analysis was performed by analyzing the mean difference between the hydrotherapy and control groups, calculated in each study by subtracting the mean change (post-intervention minus pre-intervention) in the control group from the mean change in the hydrotherapy group. 

The effect size of the intervention was studied by analyzing Cohen’s d for each of the studies, using random-effects models based on the Sidik–Jonkman method. Cohen’s d values below 0.20 indicate no effect; values between 0.21 and 0.49 indicate a small effect; values between 0.50 and 0.70 indicate a moderate effect; and values above 0.80 indicate a large effect [23]. Heterogeneity was assessed with the I^2^ statistic, and its values were classified as non-significant (0–40%), moderate (30–60%), substantial (50–90%), or considerable (75–100%) [24]; the corresponding *p*-values were also considered.

Egger’s regression asymmetry test was performed to assess publication bias, with *p* < 0.10 being considered statistically significant [25].

Meta-analyses were performed with the free and open-source statistical software Jamovi, Version 2.3.21.0, based on the R programming language.

Based on the information provided by this review, a series of premises are obtained as results that will serve to homogenize concepts about hydrotherapy during labor.

## 3. Results

**Figure 1:**
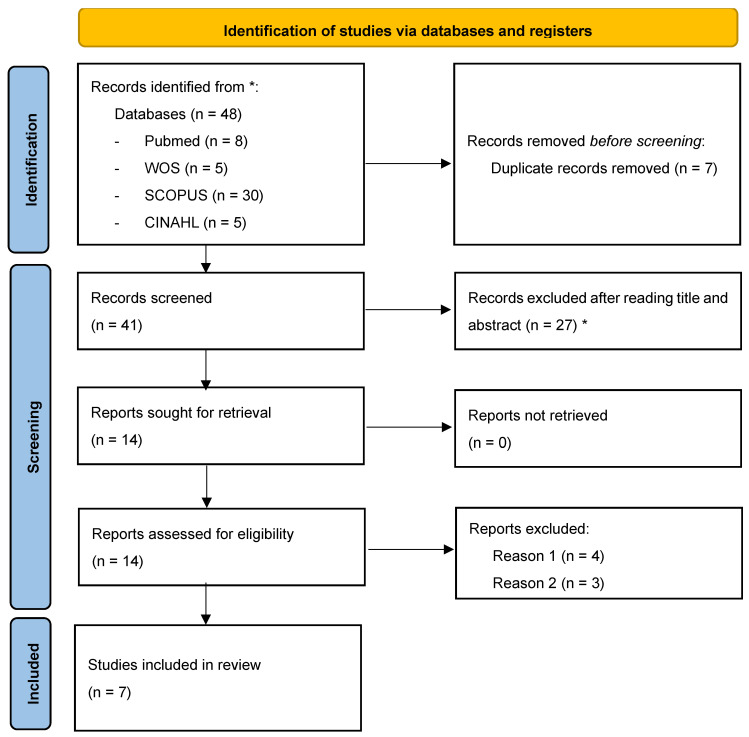
Flow diagram. * Did not meet the inclusion criteria. Reason 1: RCT protocols; Reason 2: systematic reviews of RCTs.

Seven potentially eligible studies were identified by searching electronic databases, and none were identified through other sources. Details regarding the inclusion and exclusion of studies at each stage are provided in the flow chart [26] (Figure 1).

These seven studies included a total of 840 pregnant women. The intervention groups included 417 pregnant women at term, while the control groups included 423 pregnant women.

Five articles assessed pain during the first stage of labor using the visual analog scale (VAS) as a method, and two articles assessed pain during the first stage of labor using the percentage of analgesic medication use. 

Table 1 summarizes the articles selected for the systematic review and meta-analysis.

**Figure 2:**
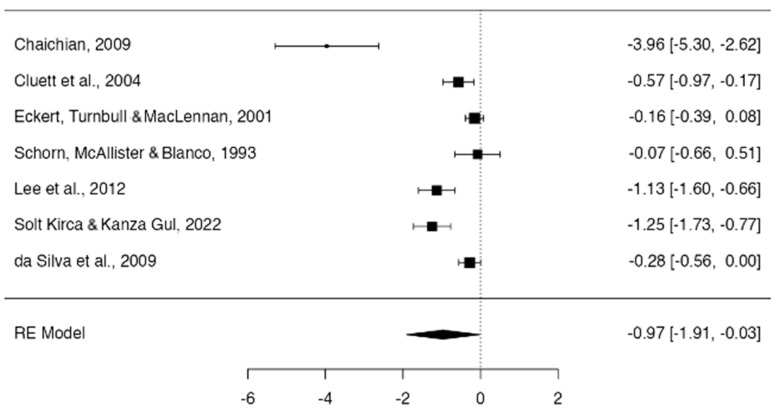
Forest plot use of hydrotherapy for pain [16,17,18,19,20,21,22].

Overall, the use of hydrotherapy reduced pain in the first stage of labor compared with the control group, showing considerable heterogeneity between studies (Pain, −0.97; 95% CI, −1.91 to −0.03; I^2^ = 97.32%, *p* < 0.001 and n = 840) (Figure 2).

**Figure 3:**
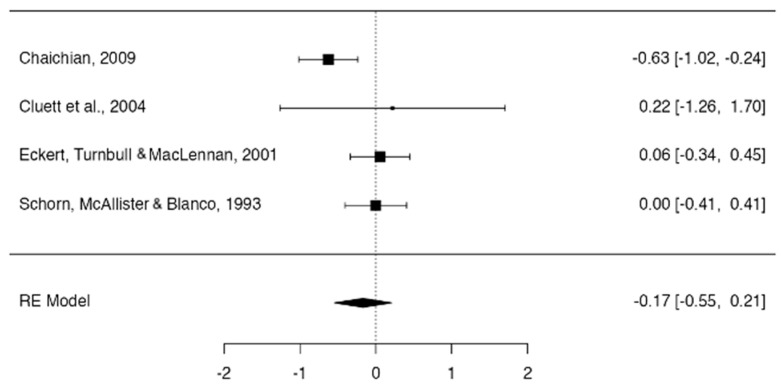
Forest plot use of hydrotherapy versus times in the first stage of labor [16,17,18,19].

The study with the largest effect size concerning pain assessment was that of Chaichian et al. [16], (d = −3.964). The studies by Lee et al. [20] and Solt Kirca and Kanza Gul [21] also presented large effects, with values of −1.127 and −1.2467, respectively. Cluett et al. [11] found a moderate effect (d = −0.5693), while da Silva et al. [22] found a small effect (d = −0.2789). Eckert, Turnbull, and MCallister [18] along with Schorn, McAllister, and Blanco [19] showed no effect on the intervention, with a Cohen’s d of less than 0.20 (−0.1552 and −0.0736, respectively) (Figure 3).

However, the use of hydrotherapy did not significantly affect the duration of the first stage of labor, with moderate heterogeneity between studies (duration of the first stage of labor −0.17; 95% CI, −0.55 to 0.21; I^2^ = 56.75%, *p* = 0.059 and n = 572) (Figure 3).

**Figure 4:**
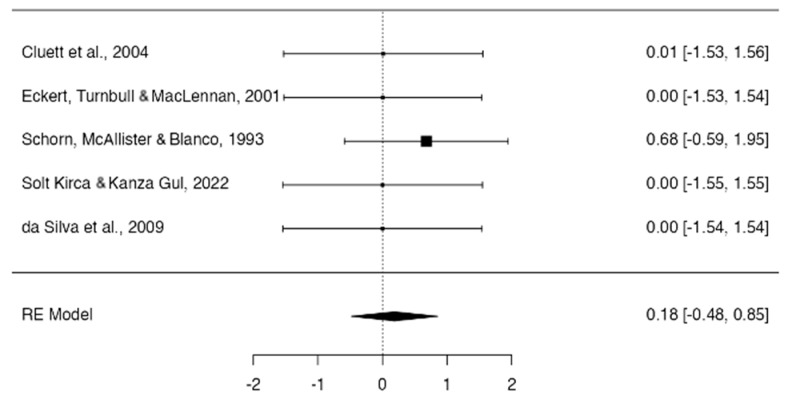
Forest plot use of hydrotherapy against the physical condition of newborns [17,18,19,21,22].

Regarding the physical condition of the newborn, it was observed that the use of hydrotherapy does not affect the physical condition of the newborn, with homogeneity in the studies (Apgar 5 min, 0.18; 95% CI, −0.48 to 0.85; I^2^ = 2.15%, *p* = 0.939 and n = 654) (Figure 4).

The assessment of methodological quality revealed that most of the information was obtained from trials with good methodological quality (Supplementary Table S2). However, all articles noted that blinding of participants, researchers, and groups was impossible due to the nature of the intervention performed during the first stage of labor.

**Figure 5:**
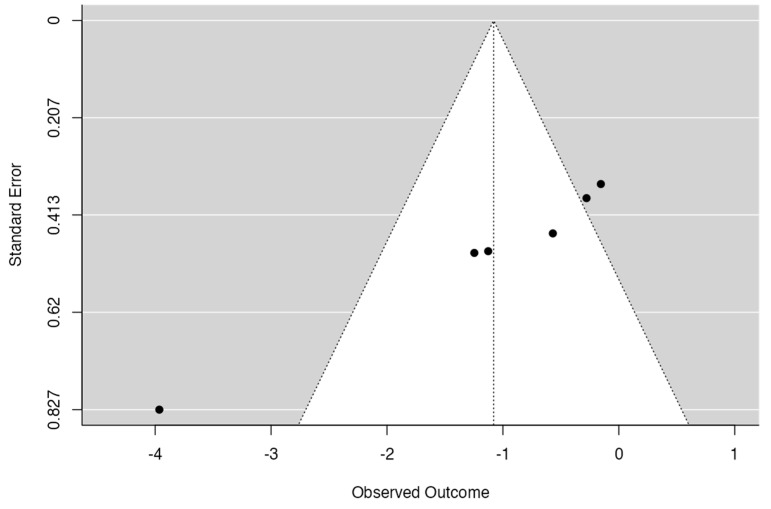
Funnel plot.

Figure 5 shows the funnel plot used to assess publication bias in the studies included in the meta-analysis. The results of the conducted tests are as follows: the fail-safe N, which indicates the number of additional studies needed to nullify the meta-analysis results, is 48 (*p* < 0.001), suggesting a high robustness of the findings. Kendall’s tau test yielded a value of −1.000 with a *p*-value of 0.003, indicating significant publication bias. Additionally, Egger’s regression produced a coefficient of −4.553 with a *p*-value of less than 0.001, confirming the presence of publication bias. These combined results suggest that although the meta-analysis shows a significant effect, the potential impact of publication bias must be considered when interpreting the findings.

## 4. Discussion

This meta-analysis has enabled us to synthesize the current relevant findings on the use of hydrotherapy during the first stage of labor. The findings of this study contribute to the evidence demonstrating significant differences in pain control during this stage between the groups using hydrotherapy and those following standard hospital procedures.

In this work, we found that the most studied outcome was pain during the first stage of labor among groups that used hydrotherapy compared with those that did not. These results indicated that hydrotherapy during labor was associated with lower pain scores in the hydrotherapy group. It should be noted that the measurement tools used in these studies varied, with most employing the visual analog pain scale [17,18,20,21,22], while some assessed pain through analgesic use [16,19]. Da Silva et al. [22] reported decreased pain in the water immersion groups compared with those that did not use hydrotherapy, combining this assessment with a behavioral pain scale between the two groups. Other studies also reported decreased pain in hydrotherapy groups at various times during the dilation phase compared with non-hydrotherapy groups that received conventional procedures such as amniotomy and oxytocin infusion [20,21]. However, no significant differences in mean scores for clinical or laboratory pain indicators were found in two articles [17,18]. A meta-analysis of the data shows that the effect size of hydrotherapy during the first stage of labor is significant compared with conventional procedures.

Although pain was perceived to be less in some studies, Eckert, Turnbull, and MCallister [18] noted that women’s use of analgesia was greater in the hydrotherapy group. When contractions intensified, they needed to exit the water and discontinue hydrotherapy. However, in general, neither group demonstrated significant differences in the amount of pharmacological analgesia administered [17,19]. Conversely, Cluett et al. [17] showed that women using water immersion had a lower rate of epidural analgesia compared with those undergoing amniotomy and oxytocin without hydrotherapy. 

In non-hydrotherapy groups, conventional management of labor, including amniotomy and oxytocin administration, was performed more frequently than in hydrotherapy groups [17,19,21].

This review found no differences in delivery types between hydrotherapy and non-hydrotherapy groups [16,17,18,19]. In a randomized controlled study by Chaichian et al. [16] involving 106 women, all women using hydrotherapy had natural birth, whereas 79.2% of those receiving conventional treatment had natural birth, although the differences were not significant. Similarly, Cluett et al. [17] found no significant differences in operative deliveries or the mean duration of the first stage of labor. Schorn et al. [19] also concluded that there were no significant differences in the duration of the first stage of labor with respect to minutes. In contrast, Chaichian et al. [16] found a significant difference in the active phase duration of the first stage of labor. The meta-analysis showed no statistically significant difference between hydrotherapy and conventional treatment in the duration of the first stage of labor (*p* = 0.059).

Neonatal outcome measures, including maternal infection rates related to neonatal infection, Apgar scores, fetal distress, or abnormal fetal cardiotocographic recordings, were similar between the two groups [16,17,19,21].

Although no differences were noted, Eckert, Turnbull, and MCallister [18] reported more use of oxygen masks and intermittent positive pressure ventilation in infants whose mothers used hydrotherapy.

Admissions to the neonatal unit were similar in both groups, with no significant differences. Cluett et al. [17] analyzed six admissions of infants born to women using water immersion and terminated in operative delivery, concluding that they experienced no subsequent problems.

Regarding maternal outcomes, Chaichian et al. [16] recorded 23% episiotomies in the non-hydrotherapy group, although tears were 12% higher in the water immersion group; however, the differences were minimal and not significant [16,17]. No differences were observed for hospital readmissions, postpartum endometritis, or postpartum pain at 24–48 h and at 8 months [17,19].

Eckert, Turnbull, and MCallister [18] assessed the birth experience, finding it more positive in the conventionally managed group in terms of relationship with staff, social support, information, choices and decisions, and satisfaction. 

Birth using hydrotherapy has been shown to be more satisfying for women, which is attributed to the freedom of movement, intimacy, and reduced labor pain intensity, all positively influencing women’s wellbeing and comfort [19]. However, studies such as Cluett et al. [17] mention this satisfaction but find no significant differences.

The main limitations of this study are closely related to the existing scientific literature on this topic. Given that hydrotherapy is an innovative technique, the current knowledge on it is limited.

Additionally, the impact of publication bias was evaluated using several statistical and visual tests. The analysis included the calculation of the fail-safe N, Kendall’s tau test, and Egger’s regression. The fail-safe N was 48 (*p* < 0.001), indicating that 48 additional studies with null effects would be needed to render the meta-analysis results non-significant. Kendall’s tau test and Egger’s regression showed values suggesting a significant presence of publication bias. These results, along with the funnel plot, indicate that although the meta-analysis results are statistically significant, the magnitude of the observed effect may be influenced by publication bias. Therefore, it is crucial to interpret the results with caution and consider this potential bias when drawing conclusions.

Future research lines have emerged from this study. A project involving four hospitals in the province of Granada will study births and pain control in pregnant women, with subsequent follow-up during the postpartum period. A control group will be established to compare results.

Additionally, the possible benefits of hydrotherapy in deliveries of pregnant women diagnosed with hypermobile Ehlers–Danlos syndrome, a rare disease, will be investigated.

## 5. Conclusions

Based on the provided information from the systematic review, several conclusions can be drawn:

Hydrotherapy as a non-pharmacological method for pain relief: The systematic review suggests that hydrotherapy during labor can serve as an effective non-pharmacological method for pain relief. This implies that it could offer an alternative or complementary approach to traditional pharmacological methods, potentially reducing the need for epidurals.

Improved coping mechanisms and satisfaction: Women who utilize hydrotherapy during labor may experience an enhanced ability to cope with pain, leading to a greater sense of control, satisfaction, and comfort. These psychological benefits can contribute positively to the overall childbirth experience.

No significant impact on labor duration or newborn health: The use of hydrotherapy does not seem to affect the duration of labor or the physical condition of the newborn. This suggests that while it provides pain relief and psychological benefits, it does not interfere with the natural progression of labor or compromise the health of the newborn.

Potential reduction in instrumental deliveries and cesarean sections: Some authors suggest that hydrotherapy may even facilitate the natural completion of labor, resulting in fewer instrumental deliveries and cesarean sections. This has significant implications for addressing concerns about the increasing rates of cesarean sections and reducing interventionism in clinical practice.

Need for further research: Despite the positive findings, there is a need for further research, particularly research focusing on the use of hydrotherapy in the second stage of labor. Additionally, the lack of reported adverse neonatal outcomes in many articles contrasts with the caution expressed by some pediatric associations, highlighting the necessity for more comprehensive studies to assess safety concerns.

Importance of correct management and training: Proper management of hydrotherapy during labor involves training and updating midwives, as well as developing clinical practice protocols and guidelines that are supported by scientific evidence. This ensures that women receive optimal care during childbirth and mitigates potential risks associated with hydrotherapy.

Overall, this systematic review suggests that hydrotherapy during labor offers promising benefits for pain relief and childbirth outcomes, but further research and proper management are necessary to fully understand its implications and ensure safe implementation.

## Figures and Tables

**Table 1:** Characteristics of included trials.

Author	Country	Method	Interventions Characteristics					Outcomes			
			Gestation Weeks (Media)	Sample Size	Distribution of the Sample	Type of Population	Average Age	Type of Intervention	Intervention Time	Pain Scale	Physical Condition of the Newborn
Chaichian, 2009 [16]	Iran	RCT	37–42 weeks	106	EG 53; CG 53	No risks	EG: 26.4 ± 5.9; CG: 27.1 ± 5.9	Warm water pools	On demand	Use of analgesics	Not reported
Cluett et al., 2004 [17]	England	RCT	EG: 284 ± 7 days; CG: 280 ± 8 days	99	EG: 49; CG: 50	Nulliparous women with dystocia and low risk of complications	EG: 26.0 ± 4.8; CG: 24.8 ± 6.0	Warm water pools	Maximum 4 h in the pool	Visual Analog Scale	Apgar 5 min
Eckert, Turnbull and MCallister, 2001 [18]	Australia	RCT	EG: 39.9 ± 1,0; CG: 39.9 ± 1,0	274	EG: 137; CG: 137	Singleton pregnancy. No risks	EG: 28.4 ± 5.4; CG: 27.2 ± 5.1	Warm water pools	On demand during the first stage of labor	Visual Analog Scale	Apgar 5 min
Schorn, McAllister and Blanco, 1993 [19]	USA	RCT	EG: 39.1 ± 1.4; CG: 39.2 ± 1.1	93	EG: 45; CG: 48	Intact membranes and no obstetric risks	EG: 21.4 ± 4.6; CG: 22.6 ± 6.1	Warm water pools	On demand	Use of analgesics	Apgar 5 min
Lee et al., 2013 [20]	Taiwan	RCT	EG: 38.91 ± 1.26; CG: 39.19 ± 1.05	80	EG: 39; CG: 41	Pregnant women with a single foetus with no risk of complications	EG: 31.44 ± 3.85; CG: 31.83 ± 4.62	Warm showers	20 min per shower	Visual Analog Scale for Pain (VASP)	Not reported
Solt and Kanza Gul, 2022 [21]	Turkey	RCT	EG: 39.2 ± 0.8; CG: 39.2 ± 0.8	80	EG: 40; CG: 40	Primiparas between 20 and 40 years old, single foetus.	EG: 28.7 ± 3.1; CG: 28.3 ± 3.2	Warm showers	20 min per shower (18 showers)	Visual Analog Scale	Apgar 5 min
da Silva et al., 2009 [22]	Brazil	RCT	EG: 39.5 ± 0.9; CG: 39.5 ± 1.1	108	EG: 54; CG: 54	Uncomplicated full-term pregnancies	EG: 19.7 ± 3.6; CG: 21.1 ± 4.1	Warm water pools	60 min	Visual Analog Scale	Apgar 5 min

## Data Availability

Data are available upon request from the corresponding author.

## Supplementary Materials

The following supporting information can be downloaded at: https://www.mdpi.com/article/10.3390/jcm13113260/s1, Supplementary Material Table S1 provides the search strategies used in each of the databases and the filters applied. Supplementary Material Table S2 provides the results of the application of the PEDro scale to each of the articles included in this meta-analysis.
