# Supplementary material for: Hydrotherapy in Pain Management in Pregnant Women: A Meta-Analysis of Randomized Clinical Trials
Source: J Clin Med. 2024 May 31;13(11):3260. doi: 10.3390/jcm13113260 (PMC11172610; doi:10.3390/jcm13113260)
Supplement: Supplementary file 1 [file jcm-13-03260-s001.zip › jcm-3025308-supplementary.pdf]

Supplementary Material

Table S1. Search strategy used in each of the databases.

Sources	Search strategy	Filters
SCOPUS	**(TITLE-ABS-KEY (immersion) OR TITLE-ABS-KEY ("Immersions" OR "Submersion" OR "Submersions") AND TITLE-ABS-KEY (labor AND stage, AND first) OR TITLE-ABS-KEY ("First Labor Stage" OR "Labor First Stage" OR "First Stage" OR "Labor" OR "Cervical Dilatation" OR "Cervical Dilatations" OR "Dilatation, Cervical" OR "Dilatations, Cervical") AND TITLE-ABS-KEY (rct) OR TITLE-ABS-KEY (randomised AND clinical AND trial))**	RCT<br>articles
PUBMED	**Search: (((immersion[MeSH Terms]) OR ("Immersions"OR"Submersion"OR"Submersions"[Title/Abstract]))) AND (((first labor stage[MeSH Terms])) OR ("First Labor Stage"OR"Labor First Stage"OR"First Stage"OR"Labor"OR"Cervical Dilatation"OR"Cervical Dilatations"OR"Dilatation, Cervical"OR"Dilatations, Cervical"[Title/Abstract]))**	RCT<br>Full text
WOS	**#3 AND #2 AND #1**<br>**(TS=("immersion")) OR TS=(“Immersions”OR”Submersion”OR”Submersions”)**<br>**(TS=("Labor Stage, First")) OR TS=(“First Labor Stage”OR”Labor First Stage”OR”First Stage”OR”Labor”OR”Cervical Dilatation”OR”Cervical Dilatations”OR”Dilatation, Cervical”OR”Dilatations, Cervical”)**<br>((TS=(RCT)) OR TS=("Randomised Clinical trial")) OR TS=("randomised clinical trial")	Document types: Clinical trial.
CINAL	**(MM "Labor Stage, First") AND (MM "Immersion")**	Enlargers - Apply Equivalent Subjects<br>Search modes - Boolean/Phase

Table S2. PEDro Scale Results.

Trial	P1	P2	P3	P4	P5	P6	P7	P8	P9	P10	TOTAL
Cluett et al., 2004 [17]	+	+	+	-	-	-	+	+	+	+	7/10
da Silva et al., 2009 [22]	+	+	+	-	-	-	-	+	+	+	6/10
Eckert, Turnbull and MacLennan, 2001 [18]	+	+	+	-	-	-	+	+	+	+	7/10
Lee et al., 2012 [20]	+	+	+	-	-	-	-	+	+	+	6/10
Schorn, McAllister and Blanco, 1993 [19]	+	-	+	-	-	-	+	-	+	+	5/10
Solt Kirca and Kanza Gul, 2022 [21]	+	+	+	-	-	-	+	-	+	+	6/10
Chaichian, 2009 [16]	+	+	+	-	-	-	-	+	+	+	6/10

Criteria: + Yes; - No. P1: Random allocation; P2: Allocation concealment; P3: Similar groups at baseline; P4: Blinding of participants; P5: Blinding of therapists; P6: Between-group blinding; P10: Point estimate and reported assessor variability; P7: Dropouts < 15%; P8: Intention-to-treat analysis; P9: Reported differences.
